# Application of a Novel Phage LPSEYT for Biological Control of *Salmonella* in Foods

**DOI:** 10.3390/microorganisms8030400

**Published:** 2020-03-12

**Authors:** Ting Yan, Lu Liang, Ping Yin, Yang Zhou, Ashraf Mahdy Sharoba, Qun Lu, Xingxing Dong, Kun Liu, Ian F. Connerton, Jinquan Li

**Affiliations:** 1 Key Laboratory of Environment Correlative Dietology, College of Food Science and Technology, Huazhong Agricultural University, Wuhan 430070, China; 2 School of Biosciences, University of Nottingham, Sutton Bonington Campus, Loughborough, Leicestershire LE12 5RD, UK; 3 College of Fisheries, Huazhong Agricultural University, Wuhan 430070, China; 4 Department of Food Technology, Faculty of Agriculture, Benha University, Benha 13518, Egypt; 5 Laboratory of Bacterial Pathogenesis and Immunology, The Rockefeller University, New York, NY 10065-6399, USA

**Keywords:** *Salmonella*, phage, characterization, genome

## Abstract

*Salmonella* is a leading cause of foodborne diseases, and in recent years, many isolates have exhibited a high level of antibiotic resistance, which has led to huge pressures on public health. Phages are a promising strategy to control food-borne pathogens. In this study, one of our environmental phage isolates, LPSEYT, was to be able to restrict the growth of zoonotic *Salmonella* *enterica* in vitro over a range of multiplicity of infections. Phage LPSEYT exhibited wide-ranging pH and thermal stability and rapid reproductive activity with a short latent period and a large burst size. Phage LPSEYT demonstrated potential efficiency as a biological control agent against *Salmonella* in a variety of food matrices, including milk and lettuce. Morphological observation, comparative genomic, and phylogenetic analysis revealed that LPSEYT does not belong to any of the currently identified genera within the *Myoviridae* family, and we suggest that LPSEYT represents a new genus, the *LPSEYTvirus*. This study contributes a phage database, develops beneficial phage resources, and sheds light on the potential application value of phages LPSEYT on food safety.

## 1. Introduction

*Salmonella* is one of the most commonly reported microorganisms associated with foodborne disease [1]. Salmonellosis in humans is closely linked with the ingestion of pathogenic *Salmonella* contaminated vegetables, fruits, and animal products. *Salmonella* contamination frequently happens during food production (e.g., harvest, packinghouse) and during food handling processes in the kitchen [2]. Although various intervention strategies have been globally developed and implemented, salmonellosis remains one of the most commonly reported anthropozoonoses. In China, the proportion of the foodborne diseases caused by *Salmonella* was estimated at 22.2% [3]. Similarly, between 2009 and 2015, the Foodborne Disease Outbreak Surveillance System (FDOSS) reported 896 outbreaks caused by *Salmonella*, accounting for 30% of total foodborne disease outbreaks in the United States [4]. *Salmonella enterica* serovar Enteritidis (*S*. Enteritidis) and *Salmonella enterica* serovar Typhimurium (*S*. Typhimurium) were the most commonly isolated serovars from outbreaks associated with foodborne salmonellosis [5]. 

Along food production chains, certain processes can potentially compromise future food safety and human health. For example, the observation that bacterial cross-contamination during transportation and storage results in high levels of multi-drug resistance, where some isolates were even resistant against triclosan [6,7]. The application of antibiotics to reduce the burden of salmonellosis in farm production has caused severe problems in respect to the rapid emergence of antibiotic-resistant strains. A study in Changchun, China, reported that 93.75% of the 48 *Salmonella* strains isolated from chicken and pork showed resistance to antibacterial agents, and 25% of the isolates were multidrug-resistant strains [8]. Under these circumstances, the development of new strategies to sustainably control food-borne pathogens to improve safety in food production is a current global need.

As an obligate, viral parasites of bacteria, bacteriophage, bind to specific receptors present on bacterial surfaces [9]. As a result, bacteriophages have extreme host-specificity so that they only infect targeted bacteria without affecting other non-targeted bacteria and to preserve the microbiota [10,11,12]. Meanwhile, bacteriophage possesses other advantages, such as self-replicating potential, rapid killing, widespread distribution, which make phage-based biocontrol an attractive alternative against bacterial pathogens in the food production chain. Phage application has been approved by Food and Drug Administration (FDA) and Food Safety and Inspection Service of the U.S. Department of Agriculture (USDA), and a variety of commercial products are available, targeting *Salmonella*, including SalmoFreshTM [13] and PhageGuard S [14]. These can be used as antimicrobial agents in food raw materials and processed products. 

Bacteriophage classification and the genetic backgrounds are critical for the application of phage therapy [15,16,17]. As of 2019, there were, in total, 69 genera of the *Myoviridae* family certified by the International Committee on Taxonomy of Viruses (ICTV) [18]. In terms of *Salmonella* phages, there were more than 180 complete genome sequences available in GenBank. For these *Salmonella* phages which belonged to *Myoviridae* family, they fell into five genera (recorded in ICTV), including *Felixo1virus*, *P2virus*, *S16virus*, *Se1virus*, and *Spn3virus* [18]. Other characterized *Salmonella* phages of the *Myoviridae* family remained unclassified. In the early days, both double-stranded or single-stranded RNA or DNA phages were classified according to the taxonomic classification, which was based on morphological similarity and the composition of nucleic acids [19,20]. With the development of sequencing technology, taxonomical classifications have become more precise on the basis of the genomes, transcriptional regime, and gene content, rather than purely based on morphological features [21,22]. Many new genera have been discovered through different methods since then. For example, Lavigne et al. reported that myoviruses could be classified by a cut-off value of 40% homologous proteins into genera [23]. The result obtained by this kind of classification method was consistent with the classification recorded in ICTV and the results from other information-based classifications [23]. In addition, the genes of terminase large subunit and major capsid protein were also used previously in an attempt to classify phages [24,25]. 

Detailed characterizations of novel *Salmonella* phage will be significant for exploring beneficial biological reagents against *Salmonella* in food safety. Genome analysis is also of use to understand the interaction mechanisms between *Salmonella* and phages and to improve the bacteriophage database and apply bacteriophages as a therapeutic approach. 

In this study, LPSEYT, a novel *Salmonella* lytic phage, was isolated and the lytic potential against *Salmonella*, morphology, pH stability, and thermal stability of LPSEYT were evaluated. As a biological control agent, the potential efficiency of LPSEYT against *Salmonella* in food matrices was also tested. Based on genome annotation, comparative genomic and phylogenetic analyses, LPSEYT is safe for application to the food chain, and it is proposed that phage LPSEYT, together with BP63 and UPF_BP2 within the database, could form the basis of a new genus *LPSEYTvirus* within the *Myoviridae* family.

## 2. Materials and Methods 

### 2.1. Bacterial Strains and Growth Conditions

Bacteria strains used in this study are listed in Table S1. Bacterial strains were grown in tryptone soya broth (Becton Dickinson, Sparks, MD, USA) at 37 °C overnight. These strains were applied for phage isolation, propagation, purification, as well as for phage lytic range and lytic activity determination in liquid cultures and in food matrix.

### 2.2. Bacteriophage Isolation

Phages were isolated from water samples obtained in Wuhan, China, based on modification of the method described by Huang et al. [26]. Water samples were centrifuged at 10,000× *g* for 10 min, followed by filtration through a 0.22-μm filter (Millipore, Ireland). The filtrate was then mixed with TSB that had been pre-inoculated with overnight-cultured *Salmonella* as host bacteria. After overnight incubation at 37 °C, the mixture was centrifuged and filtered as described above. The filtrate was detected for the presence of phages by spotting 10 μL onto double-layer agar plates where the top layer consisted of the host bacterial lawn. Any recovered phage samples were plaque purified by picking up single plaques, followed by propagation for at least three times to ensure the purity of phage isolate. The isolated phages were stored at 4 °C for further usage.

### 2.3. Salmonella Challenge Tests

The efficiency of selected phages virulence were evaluated respectively by lysing *S*. Enteritidis SGSC 4901, as published previously [27]. In brief, the experimental group was set up by infecting 100 μL freshly cultured *S*. Enteritidis SGSC 4901 (7 log_10_ CFU/mL) with 100 μL of diluted phage suspension (5 log_10_ PFU/mL) in a clear 96-well microplate (Corning Incorporated, USA). As control, 100 μL culture of *Salmonella* was mixed with 100 μL sterile TSB. The plate was maintained at 37 °C on an orbital shaker at 160 rpm and the absorbance of each sample at 600 nm (A_600_) was blanked with sterile TSB, and measured every hour for a total duration of 10 h using a Tecan microplate reader (Infinite M200 Pro, Switzerland). Candidate phages were selected based on antimicrobial activity against multiple hosts. The activities of the phages were examined against *S*. Enteritidis ATCC 13076, *S*. Enteritidis SGSC 4901, *S*. Typhimurium ATCC 13311, and *S*. Typhimurium ST-8 were further evaluated at various range of multiplicity of infections (MOIs) 0.01, 0.1, 1, 10, and 100, as described above.

### 2.4. Morphology of LPSEYT

Ten microliter of purified phage (9 log_10_ PFU/mL) was dropped onto the carbon-coated copper mesh grid and incubated at room conditions for 10 min to allow samples to bind. Any excess phage suspension was removed with filter paper after incubation. The phage was negative stained with 2% (*w/v*) phosphotungstic acid. The grid was then examined with a transmission electron microscope (TEM; Hitachi H-7000FA, Japan) at an acceleration voltage of 75 kV.

### 2.5. Phage Spot Test

Spot test was carried out according to reported methodology [28]. Overnight cultures of bacteria were mixed with TSB medium supplemented with 0.7% agar and then poured onto a pre-set TSA bottom layer to form bacterial lawn. The phage lysates (5 μL) were spotted onto the lawns, followed by incubation at 37 °C overnight.

### 2.6. Determination of Phage Adsorption Rate and One-Step Growth

Phage adsorption and one-step growth experiments were carried out according to the respective methods described by Kwiatek et al. [29] and Park et al. [30] with the following modifications. For the one-step growth experiment, briefly, 500 μL of phage suspension (5 log_10_ PFU/mL) was mixed with 500 μL of bacterial culture (8 log_10_ CFU/mL) to achieve a multiplicity of infection (MOI) of 0.001. The mixture was incubated at 37 °C for 10 min to allow phage adsorption to take place, and the sample was then centrifuged at 7000× *g* for 2 min to pellet bacteria. The supernatant was discarded to remove any free phage and the bacterial pellet with binding phage attached was gently washed twice with TSB. After washing, the pellet was re-suspended into 10 mL of TSB and incubated at 37 °C with constant shaking at 160 rpm for 180 min. Two sets of samples were collected at various time intervals. Before the titration, the second set of samples was treated with 1% chloroform to artificially lyse the cell so the appearance of first infective phage particles can be accurately measure to determine the eclipse time [31]. The titer of samples were tested using the double-layer agar plate method [32].

### 2.7. pH and Temperature Tolerance

In order to determine the influence of pH on the stability of phage, 100 μL of phage suspension (9 log_10_ PFU/mL) was individually added to a set of 900 μL TSB that have been pH pre-adjusted from 2 to 13. This mix was then incubated 37 °C for 60 min followed by phage titration. To determine the thermal stability, 100 μL of phage suspension (9 log_10_ PFU/mL) was added into 900 μL of TSB pre-warmed to various temperatures, ranging from 30 to 80 °C. Samples were incubated for 60 min and aliquots were removed for enumeration after 30 and 60 min incubation. The titer of the phage stock was measured through the double-layer agar method.

### 2.8. Phage Sequencing, Genome Annotation and Comparison

The genomic DNA of phage LPSEYT was extracted and purified according to a previous report [26]. The sequencing of the LPSEYT was performed using the HiSeq platform (Illumina, United States), and the reads obtained assembled by MicrobeTrakr plus (v0.9.1) software. Open reading frames (ORFs) of LPSEYT were predicted using MicrobeTrakr plus (v0.9.1) soft-ware, followed by annotation via BLAST and CD searching against the NCBI non-redundant database, the Conserved Domain Database (CDD), Pfam, SMART, COG, and InterPro databases. Transmembrane domains were identified using TMHMM Server v.2.0 (http://www.cbs.dtu.dk/services/TMHMM/) and signal sequences were identified using SignalP v3.0 (http://www.cbs.dtu.dk/services/SignalP-3.0/). The antimicrobial resistant genes (ARGs) in phage were tested using the database ARG–ANNOT (http://backup.mediterranee-infection.com/article.php?laref=282&titre=arg-annot). The database VFDB (http://www.mgc.ac.cn/VFs/main.htm) was used to detect any potential virulence factors in the phage genome. Whole viral nucleotide sequence similarities between phages were determined by BLASTN analysis at NCBI. Sequences of the terminase large subunit and major capsid protein of LPSEYT and of related phages were aligned employing MAFFT (v7.220), and tree reconstruction was carried out with RAxML (v8.2.4) with 500 bootstrap replicates. 

### 2.9. Infection Effect of Phage LPSEYT against Salmonella in Food

Pasteurized milk was purchased from a local supermarket. For studying the groups, 100 μL of *S*. Typhimurium ATCC 13311 was pre-cultured in logarithmic growth phase and added into 9.8 mL milk to reach a final viable count of 3 log_10_ CFU/mL. This was infected with 100 μL of diluted phage suspension to reach a final titer of 6 log_10_ PFU/mL (MOI = 1000) and 7 log_10_ PFU/mL (MOI = 10,000), respectively. Phosphate-buffered saline (PBS) was added to milk samples as negative control. Samples were either incubated at 4 °C (refrigerated conditions) or at 25 °C for 6 h. Aliquots of milk sample were removed for bacterial enumeration after 0, 2, 4, 6 h of incubation according to the method of Tomat et al. [33]. Assays were conducted in triplicate. 

Lettuce was purchased at local supermarket and stored at 4 °C. Samples were prepared as described previously [26]. The inner leaves of lettuce were washed with 75 % ethanol and allowed to air dry. The leaves were cut into disks of 1.5 cm in diameter using sterile knife, followed by UV treatment on both sides for 1 h to disinfect. The prepared samples were artificially contaminated with 10 μL of *Salmonella* inoculum in logarithmic growth phase to reach a final viable count of 3 log_10_ CFU/sample. After drying in a biosafety cabinet, 10 μL of serially diluted LPSEYT was added to a MOI of 1000 (6 log_10_ PFU/sample) or 10,000 (7 log_10_ PFU/sample), respectively. The same volumes of PBS were added to the sample as negative control. Then, samples were incubated at either 4 or 25 °C for 6 h. The lettuce samples were suspended in 1 mL PBS, followed by homogenization with sterile bars and vortexed for 30 s [34], and the bacteria was then enumerated with a 2 h interval. Experiments were conducted in triplicate.

### 2.10. Statistical Analysis

Data sets were analyzed using GraphPad Prism 6 (San Diego, CA, USA). Except as otherwise specified, data were exhibited as the mean ± SD and comparisons between data sets were carried out using the *t*-test. Bacterial counts recovered from food matrices were analyzed by the repeated measures test with a Tukey’s post-test. Different data were considered to have statistical significance when the *p*-value was <0.05. 

## 3. Results and Discussion

### 3.1. Isolation and Lytic Activity of the LPSEYT

A total of 55 phages were isolated from water samples in this study. Among all phage isolates, five phages (LPSEH, LPSEO, LPSER, LPSEX, and LPSEYT) were capable of forming clear visible plaques on all the *S*. Enteritidis and *S*. Typhimurium strains tested (Table 1). For two phages, this extended to *E. coli* strains.

**Figure 1:**
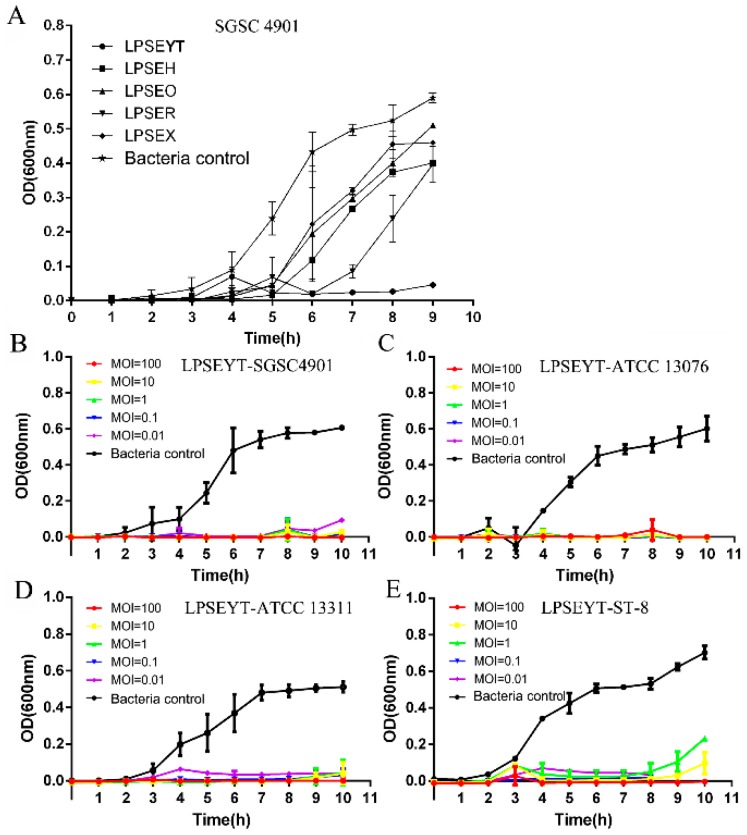
Lytic ability of isolated phages. (**A**) The antimicrobial efficacy of isolated phages against *S*. Enteritidis SGSC 4901 was compared at an MOI of 0.01. Bactericidal effects of LPSEYT against (**B**) *S*. Enteritidis SGSC 4901, (**C**) *S*. Enteritidis ATCC 13076, (**D**) *S*. Typhimurium ATCC 13311, and (**E**) *S*. Typhimurium ST-8 were evaluated at various range of multiplicity of infections (MOIs) 0.01, 0.1, 1, 10, and 100, respectively. The culture of *Salmonella* without phage was served as control.

The lytic activity of these five phages were further investigated by infecting *S*. Enteritidis SGSC 4901 in liquid cultures (Figure 1A). As shown in Figure 1A, these phages suppressed the proliferation of *Salmonella* relative to the bacteria control with a significant difference after 5 h incubation (*p* < 0.05). Greater sustained inhibitory effects were observed for LPSEYT to 9 h, while other phages permitted the proliferation of bacteria after 6 h. Hence, LPSEYT was selected for further study. To further establish the lytic capacity of LPSEYT, *S*. Enteritidis ATCC 13076, *S*. Enteritidis SGSC 4901, *S*. Typhimurium ATCC 13311, and *S*. Typhimurium ST-8 were infected by LPSEYT at multiplicity of infections (MOIs) of 0.01, 0.1, 1, 10, and 100. Restricted growth of *Salmonella* was observed for all the applied MOIs (*p* < 0.05; Figure 1B–E). Moreover, no obvious growth for *S*. Enteritidis ATCC 13076, *S*. Typhimurium ST-8, *S*. Enteritidis SGSC 4901, and *S*. Typhimurium ATCC 13311 were detected within 10 h when phage was administered at an MOI of 1000. By comparison, *Salmonella* phages FGCSSa1 and PA13076 were reported to be unable to completely lyse their hosts [35,36]. The LPSEYT exhibited vigorous lytic ability against *Salmonella* for all the MOIs tested; even when applied at an MOI of 0.01, LPSEYT was observed to restrict the growth of *Salmonella* relative to the initial inoculum over 8 h (*p* < 0.05), whereas phages fmb-p1 and PA13076 were reported to inhibit *Salmonella* propagation at MOI = 10,000 and MOI = 100, respectively [36,37]. Considering the commercial feasibility of large-scale application, a lower MOI ratio would be preferred since it would cut down the cost of production and application of the phage product.

### 3.2. Morphology and Biological Characteristics of LPSEYT

**Figure 2:**
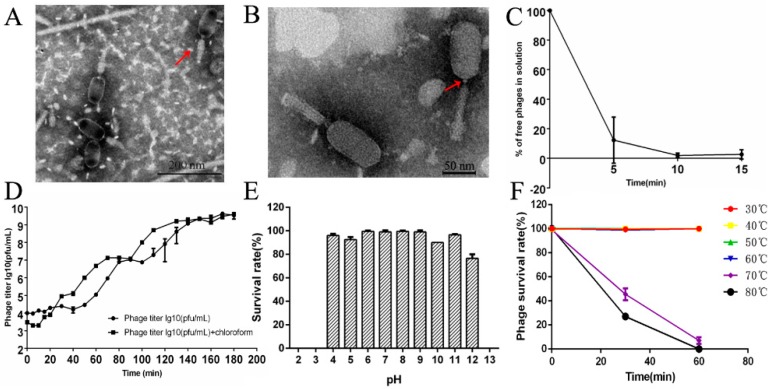
Morphology and biochemical characterization of LPSEYT. (**A**,**B**) TEM analysis of LPSEYT. (**C**) Adsorption assay of LPSEYT. (**D**) One-step growth curve of LPSEYT. (**E**) pH tolerance of LPSEYT. (**F**) Temperature tolerance of LPSEYT.

The morphological feature that distinguishes *Myoviridae* phages from other phage families is the contractile tail [19]. TEMs of phage LPSEYT showed a typical neck and a contractile tail, which suggests it is a member of the *Myoviridae* family (Figure 2A,B). The capsid of LPSEYT was measured as 90.1 ± 2.9 nm in length and 48.8 ± 3.1 nm in width (*n* = 10). According to the phage nomenclature proposed by Kropinski et al. [38], the phage name is vB_SalM-LPSEYT. Phage LPSEYT did not produce plaques on *Aeromonas hydrophila* ZYAH72, *Vibrio parahaemolyticus* ATCC 33846, *Staphylococcus aureus* ATCC 29213, and *Listeria monocytogenes* ATCC 19114. Notably, we found that phage LPSEYT was able to lyse antibiotic-resistant *Salmonella* strains listed in Table S1.

As shown in Figure 2C, the adsorption rate was more than 95% at 10 min. The one-step growth curve showed that phage LPSEYT’s lytic cycle lasted about 80 min (Figure 2D). The eclipse time, latent period, and the burst size were about 10 min, approximately 50 min and 133 ± 23 PFU/cell, respectively (Figure 2D). Compared to literature values, the latent period for LPSEYT is shorter than other myoviruses, such as *Salmonella* phage vB_SnwM_CGG4-2 (about 58 min), *Vibrio harveyi* phage VhKM4 (about 60 min), and *Stenotrophomonas maltophilia* phage φSMA5 (about 80 min) [39,40,41]. At the same time, its burst size was greater than VhKM4 (about 52 PFU/cell), phSE-1 (about 28 PFU/mL), and phSE-2 (about 53 PFU/mL) [28,40], which is likely to be a reflection of high lytic activity. The effects of pH and temperature on the stability of LPSEYT were evaluated in this study. No significant reduction in phage titer was observed after exposing the phage sample to pH 4–11 for 60 min (Figure 2E). The titer of phage LPSEYT decreased below the detection threshold (2 log PFU/mL) when exposed to strong acid (pH < 4) or alkali (pH > 11). For temperature tolerance, the survival rate of LPSEYT was almost 100% over the range 30–60 °C, while phage LPSEYT was undetectable after exposure to 80 °C for 60 min (Figure 2D). We found phage LPSEYT was more tolerant of heat and extreme pH conditions as compared with reports of other *Myoviridae* phages. Kęsik-Szeloch et al. reported that the titer of *Klebsiella pneumonia* phage KP15 decreased outside the range of pH 5 to 8 or upon exposure to 60 °C for 15 min [42]. Bao et al. reported that *Salmonella* phage PC2184 was stable over the pH range of 5 to 8 [36]. LPSEYT is more likely to retain antibacterial activity under challenging conditions, such as those experienced during food processing. 

Mitomycin C induction of the *Salmonella* hosts of LPSEYT resulted in plaque formation likely due to the prophage excision of indigenous lysogens [43]. The presence of the lysogens did not prevent superinfection from LPSEYT, which could have compromised the phages’ application against environmental *Salmonella* [44]. However, to assess whether LPSEYT could enter the lysogenic cycle, we took advantage of the phage’s ability to replicate in *E. coli* DH5α, a host that does not excise lysogens upon induction with mitomycin C. We isolated a phage resistant *E. coli* DH5α after exposure to LPSEYT, and then subjected these to mitomycin C induction. None of the resistant types produced plaques, implying they were not lysogens (Figure S1). 

### 3.3. Investigation of the Infection Effect of Phage LPSEYT against Salmonella in Food

**Figure 3:**
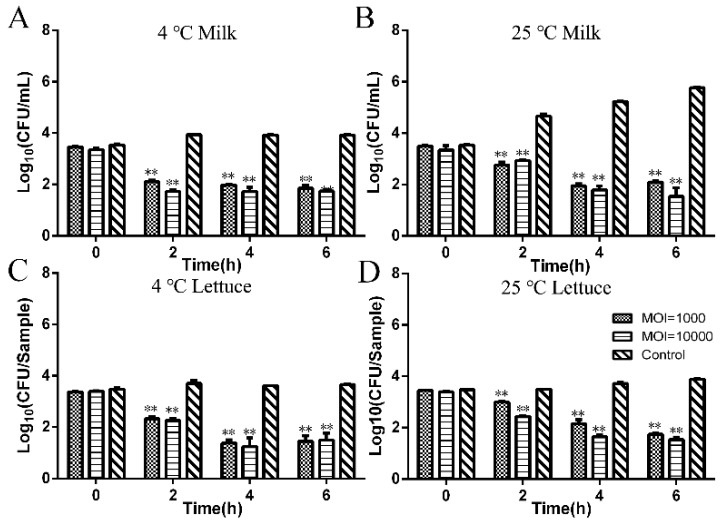
Application of LPSEYT in milk and on lettuce. Phage suspension was added into milk and onto the lettuce at an MOI of 1000 and 10,000. (**A**) LPSEYT applied in milk at 4 °C. (**B**) LPSEYT applied in milk at 25 °C. (**C**) LPSEYT applied on lettuce at 4 °C. (**D**) LPSEYT applied on lettuce at 25 °C. Data are shown as mean ± SD.

Figure 3 shows the ability of phage LPSEYT to reduce *Salmonella* in different food matrices under different temperature conditions. We found that phage LPSEYT produced significant reductions in viable *Salmonella* at both 4 °C (MOI = 1000, 10,000) and 25 °C (MOI = 1000, 10,000) regardless of the food model (*p* < 0.05). For *Salmonella*-spiked milk samples administered with phage LPSEYT at a MOI of 1000, the viable *Salmonella* count reduced by 2.07 log_10_ CFU/mL at 4 °C (Figure 3A) and 3.67 log_10_ CFU/mL at 25 °C (Figure 3B), relative to the phage-excluded control. When administered at an MOI of 10,000, phage LPSEYT reduced the *Salmonella* viable count by 2.19 log_10_ CFU/mL at 4 °C (Figure 3A) and 4.22 log_10_ CFU/mL at 25 °C (Figure 3B) after 6 hours of incubation. The antibacterial effect of LPSEYT was more durable than PA13076, as characterized by Bao et al. [36], which inhibited bacteria in milk for up to 5 h. Using a lettuce contamination model, phage LPSEYT applied at MOI = 10,000 produced a reduction in the viable count of *Salmonella* by 2.2 log_10_ CFU/sample at 4 °C (Figure 3C) and 2.34 log_10_ CFU/sample at 25 °C (Figure 3D) after 6 hours of incubation. It is clear that the antimicrobial effect of bacteriophage LPSEYT was greater at 25 than 4 °C. This is not surprising considering phage infection is dependent on the replication of their bacterial host. It would be difficult for phages to replicate effectively given the reduced metabolism of their bacterial host in refrigerated conditions. 

Previous studies have shown that the application of both phage and endolysin derived from phage exhibited greater antimicrobial activity and/or wider spectrum of lysis in comparison to the phage applied alone [45]. Therefore, future studies may focus on the expression of the LPSEYT’s endolysin as a bacterial inhibitor, and a combination of LPSEYT and other natural resources to further enhance the antibacterial effect on different food matrices.

### 3.4. Sequencing and Bioinformatics Analysis

**Figure 4:**
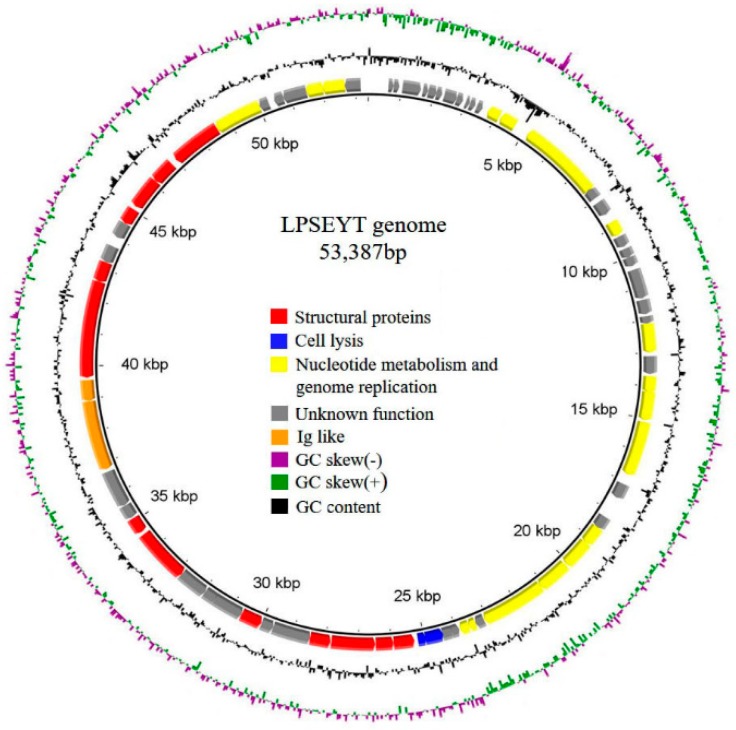
Genomic organization of LPSEYT. Patterns are divided into four circles: the full length of the genome is indicated in the first circle; the open reading frame is indicated in the second circle, and ORFs are transcribed in the clockwise or the counterclockwise direction; GC content is indicated in the third circle; while on the fourth circle, GC skew of G-C/G+C is indicated as green and purple, and green means that the value of GC skew is greater than 0 and purple means that the value is less than 0. The open reading frames marked with red, blue, yellow and orange indicate genes encoding structural proteins, cell lysis proteins, nucleotide metabolism and genome replication proteins, and Ig-like proteins, respectively; ORFs with homology to unannotated proteins or hypothetical proteins in the database are indicated in grey.

The complete genome of phage LPSEYT is a double-stranded DNA of 53,387 bp with an overall G+C content of 46.01%. Seventy-three ORFs were identified with ATG and GTG as initiation codons. Thirty-four predicted proteins showed significant homology to proteins with known functions in NCBI database (Table S2). Homologues of the remaining thirty-nine ORFs were uncharacterized proteins. Of the coding functions identified, these could classified as structural proteins and proteins involved in cell lysis, nucleotide metabolism, and genome replication (Figure 4). The absence of integrases, repressors, transposases, excisionases, antimicrobial-resistant genes, and virulence factor homologues within the genome supported that phage LPSEYT is a virulent phage and is suitable for safe application. Interestingly, within phage LPSETY’s genome, we identified two proteins containing Ig-like domains, which have been reported in association with phage that binds to human intestinal mucosa and helps the body to fight off bacterial infections [46]. However, the exact function of Ig-like proteins between phage and human immune system remains unclear. The lysis cassette of LPSEYT also drew our attention. The holin is nominally a class III holin but the topology of the transmembrane domain (TMD) of this holin is predicted to be different to the prototype class III holin of bacteriophage T4 [47,48]. Although previously not evident in *Salmonella* phages, the topology of LPSEYT’s holin is similar to that reported for *Escherichia coli* phage KBNP1315 and mycobacteriophage Ms6 [49,50]. The endolysin of LPSEYT’s lysis cassette was predicted to have a transmembrane helix in the N-terminal region as the terminal signal–anchor–release (SAR) domain. The characteristics of the N-terminal region of endolysins are important to the mechanism of lysis. It was reported that the N-terminal SAR domain is associated with the permeabilization of endolysin for Gram-negative species so that such endolysins could exhibit exogenous antibacterial activity without outer membrane permeabilization [51]. This could mean that the endolysin of LPSEYT might possess exogenous antibacterial activity.

After search of the NCBI database, 11 phages with similar nucleotide sequences of LPSEYT were found with BLASTN scores of more than 200, including 4 *Salmonella* phages (Table 2). Comparative genomics analysis indicated that phages LPSEYT, UPF_BP2, and BP63 are closely related. Although *Salmonella* phages UPF_BP2 and BP63 showed more than 90% query coverage with LPSEYT, characterization of these phages has not been reported (Table 2). The query coverages between the rest of the phages and LPSEYT were less than 2%. Therefore, it was proposed that LPSEYT is a novel phage and might belong to an unknown genus.

### 3.5. Classification of Lytic Phage LPSEYT and a New Genus LPSEYTvirus Can Be Proposed

The established method for classifying myoviruses into a genus is 40% of proteins matching within a 75-bit score by BLASTP [23]. To further investigate the genus of phage LPSEYT, protein homology analysis was performed. In this study, 16 phages that showed more than four homologous proteins with phage LPSEYT were collected and are recorded in Table 3. The result showed that the LPSEYT shared 70 (95%) homologous proteins with phage BP63, 47 (64%) homologous proteins with phage UPF_BP2, and less than 7 homologous proteins with any other phage. The homology analysis of phages BP63 and UPF_BP2 was also performed and, similarly, they could not be classified into any known genus. These results strongly indicate that LPSEYT, BP63, and UPF_BP2 have close ties and can be distinguished from other phage genus. Therefore, LPSEYT, BP63, and UPF_BP2 belong to a new phage genus and can be proposed as *LPSEYTvirus*.

**Figure 5:**
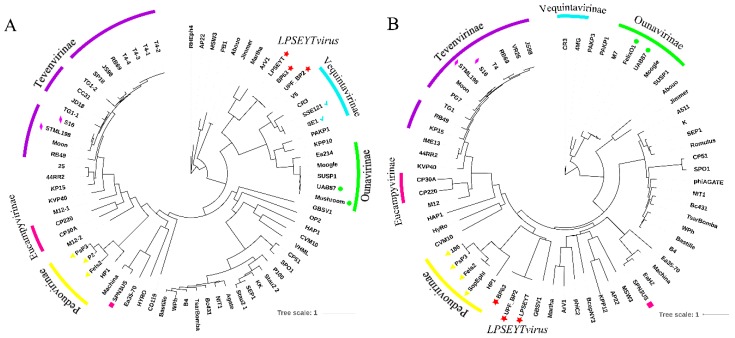
Phylogenetic analysis of selected phages and phages of the new proposed genus *LPSEYTvirus* based on (**A**) terminase large subunit and (**B**) major capsid protein. The relative distances of each main branch are shown in the figure. The arc with the same color is used to exhibit phages that are classified as the same subfamily. The triangle, rhombus, square, checkmark, and roundness represent the genera *P2virus*, *S16virus*, *Spn3virus*, *Se1virus,* and *Felixo1virus*, respectively. The star represents the phages of the new proposed genus *LPSEYTvirus* in this study.

To further validate that the proposed new genus *LPSEYTvirus* was distinguishable and not overlapping with any established genera of *Myoviridae* recognized by ICTV, phylogenetic analysis was carried out based on the amino acid sequences of the terminase large subunit and major capsid proteins, which are frequently used to classify phages [24,25]. Since organization and basic features of the terminase large subunit are generally well-conserved in tailed phages, phages with the same genus would expect to have a clustering of their terminase large subunit [63]. The major capsid protein is also suitable for phylogenetic analysis because it is relatively conserved, with little or no evidence of horizontal swapping within the phage head gene regions [64]. Protein sequences of the terminase large subunit and major capsid protein were extracted from phages of the proposed new genus *LPSEYTvirus*, representative phages of different genera of *Myoviridae* family (Table S3), and all *Salmonella* phages of *Myoviridae* family recorded by ICTV (Table 4). Different subfamilies or genera were shown in the phylogenetic tree (Figure 5A,B). The phylogenetic analysis confirmed the taxonomy listed in Table 4 and Table S3, in which partial myoviruses have been grouped into six subfamilies and *Salmonella* phages have been classified to five phage genera. As expected, the LPSEYT, BP63, and UPF_BP2 were closely clustered, distinguishing them from other *Salmonella* phages and other genera of the *Myoviridae* (Figure 5A,B). Thus, this finding reinforces the proposal that LPSEYT, BP63, and UPF_BP2 belong to a new genus, *LPSEYTvirus*.

We believe that with more isolates and genomic sequences becoming available within this newly characterized genus, we will gain a better understanding of the biological functions and properties of the proposed new genus.

## 4. Conclusions

In this study, we isolate and characterize a new lytic phage, LPSEYT, of the *Myoviridae* family. Phage LPSEYT exhibits high stability against a wide range of pH and temperatures and exhibits a potent anti-*Salmonella* effect that makes it a promising agent for application in food production. Genome analysis indicates that LPSEYT contains no virulence genes, making it suitable for development for safe use in the food industry. Comparative analysis of the nucleotide sequence and protein-coding content suggests that LPSEYT is a representative of a new genus of the *Myoviridae*, the *LPSEYTvirus*.

## Figures and Tables

**Table 1:** Lytic range of phages LPSEH, LPSEO, LPSER, LPSEX, and LPSEYT.

Bacterial	Infectivity of Phage				
	LPSEH	LPSEO	LPSER	LPSEX	LPSEYT
*S.* Enteritidis ATCC 13076	+	+	+	+	+
*S.* Enteritidis SJTUF 10978	+	+	+	+	+
*S.* Enteritidis SJTUF 10984	+	+	+	+	+
*S.* Enteritidis LSE4 (LK5)	+	+	+	+	+
*S.* Enteritidis SGSC 4901	+	+	+	+	+
*S.* Typhimurium ATCC 14028	+	+	+	+	+
*S.* Typhimurium ATCC 13311	+	+	+	+	+
*S.* Typhimurium LST2 (ST-8)	+	+	+	+	+
*S.* Typhimurium LST4 (UK-1)	+	+	+	+	+
*S.* Typhimurium LST8 (SL1344)	+	+	+	+	+
*S.* Typhimurium SGSC 4903	+	+	+	+	+
*S.* Typhi LSX1 (CT18)	+	+	–	+	+
*S.* Typhi LSX2 (Ty2)	+	+	–	+	+
*S.* pullorum CVCC 519	+	+	+	+	+
*S.* pullorum CVCC 534	+	–	+	–	+
*Escherichia coli* LEC4 (DH5α)	–	–	+	–	+
*Escherichia coli* LEC6 (BL21)	–	–	–	–	–
*Escherichia coli* LEC10 (D41)	–	–	–	–	+
*Escherichia coli* ATCC 10798	–	–	–	–	+

**Table 2:** Comparison of phages with BLASTN scores of more than 200 against the LPSEYT genome.

Phage	Country	Accession	Genome Length (bp)	E Value	BLASTN Score	Query Cover	Identify	Ref.
*Salmonella* phage UPF_BP2	Brazil	KX826077.1	54,894	0	9.0 × 10^4^	96%	97%	unpublished
*Salmonella* phage BP63	Canada	KM366099.1	52,437	0	8.6 × 10^4^	98%	98%	unpublished
*Erwinia* phage vB_EamM-M7	Switzerland	HQ728263.1	84,694	2 × 10^−14^	322	2%	65%	[52]
*Erwinia* phage vB_EamM_Asesino	USA	KX397364.1	246,291	1 × 10^−16^	283	1%	66%	unpublished
*Cronobacter* phage vB_CsaP_GAP52	Canada	JN882286.1	76,631	3 × 10^−69^	280	1%	69%	unpublished
*Acinetobacter* phage SH-Ab 15497	China	MG674163.1	43,420	1 × 10^−30^	276	1%	66%	unpublished
*Cronobacter* phage vB_CsaP_Ss1	Ireland	KM058087.1	42,205	1 × 10^−17^	267	1%	67%	[53]
*Salmonella* phage SE131	South Korea	MG873442.1	89,910	4 × 10^−55^	233	1%	67%	unpublished
*Salmonella* phage 7-11	Canada	HM997019.1	89,916	4 × 10^−54^	230	1%	67%	[54]
*Escherichia* phage vB_EcoM_PHB05	China	MF805809.1	147,659	5 × 10^−15^	211	<1%	72%	[55]
*Enterobacteria* phage ECGD1	China	KU522583.1	146,647	3 × 10^−12^	204	<1%	71%	unpublished

**Table 3:** Phages with homology genes with *Salmonella* phage LPSEYT by BLASTP.

Phage name	Phage Family	Genus	Genome Length (kb)	Accession	Homologous Genes with LPSEYT *^a^*	Homologous Proteins Rate	Reference
*Salmonella* phage BP63	*Caudovirales*	unclassified	52.437	NC_031250	70	95.89%	unpublished
*Salmonella* phage UPF_BP2	*Caudovirales*	unclassified	54.894	KX826077	47	64.38%	unpublished
*Paracoccus* phage vB_PmaS_IMEP1	*Siphoviridae*	unclassified	42.093	NC_026608	7	9.59%	unpublished
*Achromobacter* phage JWX	*Siphoviridae*	*Steinhofvirus*	49.714	NC_028768	4	5.48%	[56]
*Acinetobacter* phage SH-Ab 15497	unclassified	unclassified	43.42	MG674163	4	5.48%	unpublished
*Burkholderia* phage BcepGomr	*Siphoviridae*	unclassified	52.414	NC_009447	4	5.48%	unpublished
*Burkholderia* phage KL1	*Siphoviridae*	*Septimatrevirus*	42.832	NC_018278	4	5.48%	[57]
*Pantoea* phage vB_PagS_Vid5	*Siphoviridae*	*Vidquintavirus*	61.437	MG948468	4	5.48%	unpublished
*Pseudomonas* phage 73	*Siphoviridae*	*Septimatrevirus*	42.999	NC_007806	4	5.48%	[58]
*Pseudomonas* phage JG054	*Siphoviridae*	*Nipunavirus*	57.839	KX898400	4	5.48%	unpublished
*Pseudomonas* phage NP1	*Siphoviridae*	*Nipunavirus*	58.566	NC_031058	4	5.48%	unpublished
*Pseudomonas* phage PaMx42	*Siphoviridae*	*Septimatrevirus*	43.225	NC_028879	4	5.48%	[59]
*Pseudomonas* phage vB_PaeS_SCH_Ab26	*Siphoviridae*	*Septimatrevirus*	43.056	HG962376	4	5.48%	[60]
*Stenotrophomonas* phage vB_SmaS-DLP_2	*Siphoviridae*	*Septimatrevirus*	42.593	NC_029019	4	5.48%	[61]
*Vibrio* phage VpKK5	*Siphoviridae*	unclassified	56.637	NC_026610	4	5.48%	[62]
*Xanthomonas* phage XAJ2	unclassified	unclassified	49.241	KU197014	4	5.48%	unpublished

*^a^* The number of homologous proteins between LPSEYT and other phages.

**Table 4:** Overview of International Committee on Taxonomy of Viruses (ICTV) recorded genus of *Myoviridae* that infect *Salmonella* Spp.

Family	Subfamily	Genus	Phage	Accession	Country	Genome Length (kb)
*Myoviridae*	unknown	*Spn3virus*	*Salmonella virus SPN3US*	NC_027402	South Korea	240.413
*Myoviridae*	*Ounavirinae*	*Felixo1virus*	*Salmonella virus FelixO1*	AF320576	USA	86.155
*Myoviridae*	*Ounavirinae*	*Felixo1virus*	*Salmonella virus HB2014*	unknown	unknown	unknown
*Myoviridae*	*Ounavirinae*	*Felixo1virus*	*Salmonella virus Mushroom*	KP143762	USA	87.709
*Myoviridae*	*Ounavirinae*	*Felixo1virus*	*Salmonella virus UAB87*	JN225449	Espanya	87.603
*Myoviridae*	*Peduovirinae*	*P2virus*	*Salmonella virus Fels2*	NC_010463	USA	33.693
*Myoviridae*	*Peduovirinae*	*P2virus*	*Salmonella virus PsP3*	NC_005340	USA	30.636
*Myoviridae*	*Peduovirinae*	*P2virus*	*Salmonella virus SopEphi*	unknown	unknown	unknown
*Myoviridae*	*Tevenvirinae*	*S16virus*	*Salmonella virus S16*	NC_020416	Switzerland	160.221
*Myoviridae*	*Tevenvirinae*	*S16virus*	*Salmonella virus STML198*	NC_027344	USA	158.099
*Myoviridae*	*Vequintavirinae*	*Se1virus*	*Salmonella virus SE1*	GU070616	Portugal	145.964
*Myoviridae*	*Vequintavirinae*	*Se1virus*	*Salmonella virus SSE121*	NC_027351	USA	147.745

## Supplementary Materials

Supplementary materials can be found at https://www.mdpi.com/2076-2607/8/3/400/s1.
