# Supplementary material for: Application of a Novel Phage LPSEYT for Biological Control of Salmonella in Foods
Source: Microorganisms. 2020 Mar 12;8(3):400. doi: 10.3390/microorganisms8030400 (PMC7142823; doi:10.3390/microorganisms8030400)
Supplement: Supplementary file 1 [file microorganisms-08-00400-s001.zip › Supplementary figure 2.docx]

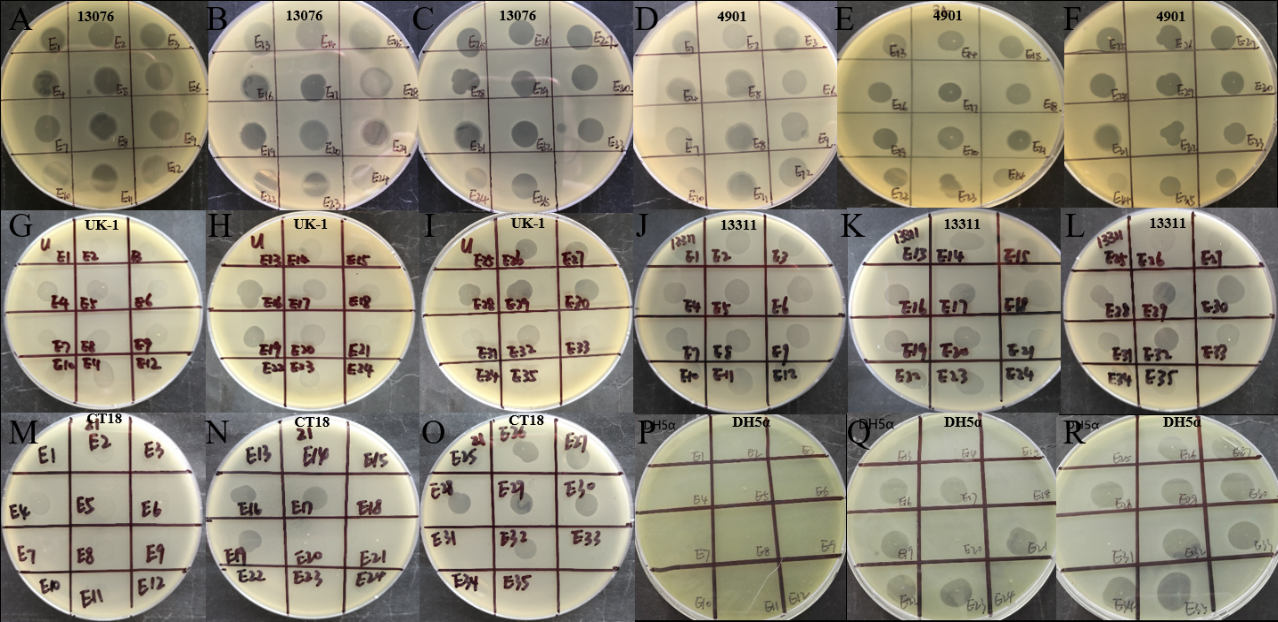


**Supplementary Figure 2.** The representative plagues of LPSEYT host range. LPSEH, LPSEO, LPSER, LPSEX and LPSEYT were represented by E8, E9, E22, E24 and E34, respectively.
