# Supplementary material for: Application of a Novel Phage LPSEYT for Biological Control of Salmonella in Foods
Source: Microorganisms. 2020 Mar 12;8(3):400. doi: 10.3390/microorganisms8030400 (PMC7142823; doi:10.3390/microorganisms8030400)
Supplement: Supplementary file 1 [file microorganisms-08-00400-s001.zip › Supplementary table 1.docx]

**Supplementary Table 1**

List of bacterial strains that were used in this study.

| Bacterial isolates | Source of strains | Reference |
| --- | --- | --- |
| *Salmonella enterica* serovar Enteritidis |  |  |
| ATCC 13076 | American Type Culture Collection | [1] |
| SJTUF 10978 | Shanghai Jiao Tong University | [1] |
| SJTUF 10984 | Shanghai Jiao Tong University | [1] |
| LSE4 (LK5) | Lab Stock | [2] |
| SGSC 4901 | Salmonella Genetic Stock Centre | [3] |
| *Salmonella enterica* serovar Typhimurium |  |  |
| ATCC 14028 | American Type Culture Collection | [1] |
| ATCC 13311 | American Type Culture Collection | [1] |
| LST2 (ST-8) | Clinical isolate | [2] |
| LST4 (UK-1) | Lab Stock | [3] |
| LST8 (SL1344) | Lab Stock | [3] |
| SGSC 4903 | Salmonella Genetic Stock Centre | [3] |
| *Salmonella enterica* serovar Typhi |  |  |
| LSX1 (CT18) | Lab Stock | [4] |
| LSX2 (Ty2) | Lab Stock | [4] |
| Salmonella *enterica* serovar pullorum |  |  |
| CVCC 519 | China Veterinary Culture Collection Center | [3] |
| CVCC 534 | China Veterinary Culture Collection Center | [1] |
| *Escherichia coli* |  |  |
| LEC4 (DH5α) | TransGen Biotech | [1] |
| LEC6 (BL21) | TransGen Biotech | [3] |
| LEC10 (D41) | Lab Stock | [5] |
| ATCC 10798 | American Type Culture Collection | [6] |
| *Aeromonas hydrophila* |  |  |
| ZYAH72 | Lab Stock | [3] |
| *Vibrio parahaemolyticus* |  |  |
| ATCC 33846 | American Type Culture Collection | [7] |
| *Staphylococcus aureus* |  |  |
| ATCC 29213 | American Type Culture Collection | [3] |
| *Listeria monocytogenes* |  |  |
| ATCC 19114 | American Type Culture Collection | [8] |
| Antibiotic-resistant strains |  |  |
| *Salmonella enterica* serovar Enteritidis |  |  |
| LSE6 | Lab Stock | [3] |
| LSE7 | Lab Stock | [3] |
| LSE8 | Lab Stock | [3] |
| LSE9 | Lab Stock | [3] |
| LSE10 | Lab Stock | [3] |
| LSE11 | Lab Stock | [3] |
| LSE12 | Lab Stock | [3] |
| LSE13 | Lab Stock | [3] |
| LSE14 | Lab Stock | [9] |
| LSE15 | Lab Stock | [3] |
| *Salmonella enterica* serovar Typhimurium |  |  |
| LST11 | Lab Stock | [3] |
| LST12 | Lab Stock | [3] |
| LST13 | Lab Stock | [3] |
| LST14 | Lab Stock | [3] |
| LST15 | Lab Stock | [3] |
| LST16 | Lab Stock | [3] |
| LST17 | Lab Stock | [3] |

REFERENCE

1. Huang, C.; Shi, J.; Ma, W.; Li, Z.; Wang, J.; Li, J.; Wang, X. Isolation, characterization, and application of a novel specific *Salmonella* bacteriophage in different food matrices. *Food Res. Int.* **2018**, *111*, 631–641.

2. Blondel, C.J.; Yang, H.J.; Castro, B.; Chiang, S.; Toro, T.C.; Zaldívar, M.; Contreras, I.; Andrews-Polymenis, H.L.; ASantiviago, C.A. Contribution of the type VI secretion system encoded in SPI-19 to chicken colonization by *Salmonella enterica* serotypes Gallinarum and Enteritidis. *Plos One* **2010**, *5*, 1–10.

3. Islam, M.; Zhou, Y.; Liang, L.; Nime, I.; Liu, K.; Yan, T.; Wang, X.; Li, J. Application of a phage cocktail for control of *Salmonella* in foods and reducing biofilms. *Viruses* **2019**, *11*, 1–19.

4. Deng, W.; Liou, S.R.; Plunkett III, G.; Mayhew, G.F.; Rose, D.J.; Burland, V.; Kodoyianni, V.; Schwartz, D.C.; Blattner, F.R. Comparative genomics of *Salmonella enterica* serovar Typhi strains Ty2 and CT18. *J. Bacteriol.* **2003**, *185*, 2330–2337.

5. Xu, T.; Ying, J.; Yao, X.; Song, Y.; Ma, P.; Bao, B.; Jiang, W.; Wu, X.; Tou, H.; Li, P.; et al. Identification and characterization of two novel bla KLUC resistance genes through large-scale resistance plasmids sequencing. *Plos One* **2012**, *7*, 1–7.

6. Dimitrova, D.; Engelbrecht, K.C.; Putonti, C.; Koenig, D.W.; Wolfe, A.J. Draft genome sequence of *Escherichia coli* K-12 (ATCC 10798). *Genome Announc* **2017**, *5*, 1–2.

7. Sun, X.; Xu, Q.; Pan, Y.; Lan, W.; Zhao, Y.; Wu, V.C.H. A loop-mediated isothermal amplification method for rapid detection of *Vibrio parahaemolyticusin* seafood. *Ann. Microbiol.* **2012**, *62*, 263–271.

8. Wei, S.; Daliri, E.B.; Chelliah, R.; Park, B.; Lim, J.; Baek, M.; Nam, Y.; Seo, K.; Jin, Y.; Oh, D. Development of a multiplex real‐time PCR for simultaneous detection of *Bacillus cereus*, *Listeria monocytogenes*, and *Staphylococcus aureus* in food samples. *J. Food Saf.* **2019**, 1–7.

9. Li, J.Q. “*Salmonella* enteritidis phage LPSE28 and its application in food” C.N. Patent 108546685 A, issued September 18, 2018.
