# Supplementary material for: Application of a Novel Phage LPSEYT for Biological Control of Salmonella in Foods
Source: Microorganisms. 2020 Mar 12;8(3):400. doi: 10.3390/microorganisms8030400 (PMC7142823; doi:10.3390/microorganisms8030400)
Supplement: Supplementary file 1 [file microorganisms-08-00400-s001.zip › Supplementary table 2.docx]

**Supplementary Table 2**. LPSEYT genome annotation.

| ORF | Strand | Start position | End position | Function |
| --- | --- | --- | --- | --- |
| 1 | + | 613 | 762 | hypothetical protein |
| 2 | + | 767 | 940 | hypothetical protein |
| 3 | + | 1036 | 1593 | hypothetical protein |
| 4 | + | 1658 | 1816 | hypothetical protein |
| 5 | + | 1822 | 2079 | hypothetical protein |
| 6 | + | 2076 | 2249 | hypothetical protein |
| 7 | + | 2318 | 2707 | hypothetical protein |
| 8 | + | 2707 | 2907 | hypothetical protein |
| 9 | + | 3000 | 3125 | hypothetical protein |
| 10 | + | 3137 | 3304 | hypothetical protein |
| 11 | + | 3360 | 3539 | hypothetical protein |
| 12 | - | 4107 | 3718 | endonuclease |
| 13 | - | 4648 | 4148 | homing endonuclease |
| 14 | - | 7639 | 5105 | primase |
| 15 | - | 7946 | 7614 | hypothetical protein |
| 16 | + | 8100 | 8537 | hypothetical protein |
| 17 | + | 8819 | 9268 | putative DNA binding protein |
| 18 | + | 9313 | 9654 | hypothetical protein |
| 19 | + | 9723 | 10013 | hypothetical protein |
| 20 | + | 10010 | 10300 | hypothetical protein |
| 21 | + | 10415 | 11320 | hypothetical protein |
| 22 | + | 11369 | 11815 | hypothetical protein |
| 23 | + | 11876 | 12106 | hypothetical protein |
| 24 | + | 12106 | 12990 | AAA family ATPase |
| 25 | + | 13098 | 13643 | hypothetical protein |
| 26 | + | 13705 | 14190 | deoxycytidylate deaminase |
| 27 | + | 14174 | 15052 | YqaJ-like viral recombinase domain-containing protein |
| 28 | + | 15111 | 16781 | superfamily II helicase |
| 29 | + | 17096 | 17563 | hypothetical protein |
| 30 | + | 18239 | 18637 | hypothetical protein |
| 31 | + | 18624 | 19217 | Cof hydrolase |
| 32 | + | 19217 | 20101 | putative thymidylate synthase |
| 33 | + | 20132 | 21127 | DNA polymerase beta subunit |
| 34 | + | 21127 | 23094 | DNA polymerase |
| 35 | + | 23110 | 23385 | hypothetical protein |
| 36 | + | 23385 | 23609 | DnaK suppressor protein |
| 37 | + | 23609 | 23887 | ryanodine receptor |
| 38 | - | 24161 | 23904 | hypothetical protein |
| 39 | - | 24435 | 24106 | hypothetical protein |
| 40 | - | 24989 | 24414 | lysozyme |
| 41 | - | 25194 | 24934 | holin |
| 42 | - | 25800 | 25297 | putative tail fiber assembly protein |
| 43 | - | 25927 | 25733 | putative tail fiber assembly protein |
| 44 | - | 26473 | 25931 | putative tail fiber assembly protein |
| 45 | - | 27817 | 26483 | putative tail fiber protein |
| 46 | - | 28463 | 27810 | hypothetical protein |
| 47 | - | 29628 | 28456 | hypothetical protein |
| 48 | - | 29999 | 29628 | hypothetical protein |
| 49 | - | 30657 | 30010 | baseplate protein |
| 50 | - | 31616 | 30657 | hypothetical protein |
| 51 | - | 31950 | 31558 | hypothetical protein |
| 52 | - | 32846 | 31950 | hypothetical protein |
| 53 | - | 34507 | 32843 | putative tail tape measure protein |
| 54 | - | 35098 | 34670 | Phage tail assemby chaperone protein |
| 55 | - | 35548 | 35117 | hypothetical protein |
| 56 | - | 36699 | 35560 | hypothetical protein |
| 57 | - | 38915 | 36786 | putative Ig-like domain-containing protein |
| 58 | - | 39565 | 38927 | bacterial Ig-like domain family protein |
| 59 | - | 42590 | 39633 | tail protein |
| 60 | - | 43213 | 42590 | putative tail fiber protein |
| 61 | - | 43740 | 43213 | hypothetical protein |
| 62 | - | 44529 | 44041 | hypothetical protein |
| 63 | - | 45029 | 44529 | virion protein |
| 64 | - | 46111 | 45134 | major capsid protein |
| 65 | - | 46828 | 46115 | scaffold protein |
| 66 | - | 48568 | 47006 | portal protein |
| 67 | - | 50018 | 48570 | terminase large subunit |
| 68 | - | 50312 | 50022 | hypothetical protein |
| 69 | - | 50780 | 50490 | hypothetical protein |
| 70 | - | 51499 | 50780 | hypothetical protein |
| 71 | - | 52002 | 51496 | thymidylate kinase |
| 72 | - | 52673 | 51999 | dihydrofolate reductase |
| 73 | - | 53151 | 52666 | hypothetical protein |
