# Supplementary material for: Application of a Novel Phage LPSEYT for Biological Control of Salmonella in Foods
Source: Microorganisms. 2020 Mar 12;8(3):400. doi: 10.3390/microorganisms8030400 (PMC7142823; doi:10.3390/microorganisms8030400)
Supplement: Supplementary file 1 [file microorganisms-08-00400-s001.zip › Supplementary table 3.docx]

**Supplementary Table 3**

Representative phage from different phage genus of *Myoviridae* family recorded by ICTV and used in this study.

| Phage family | Subfamily | Genus | Phage name | Accession | Country | Genome length(kb) |
| --- | --- | --- | --- | --- | --- | --- |
| *Myoviridae* | unknown | *Ap22virus* | *Acinetobacter virus AP22* | HE806280 | RUSSIA | 46.387 |
| *Myoviridae* | unknown | *Secunda5virus* | *Aeromonas virus 25* | DQ529280 | USA | 161.475 |
| *Myoviridae* | unknown | *Secunda5virus* | *Stenotrophomonas virus IME13* | JX306041 | China | 162.327 |
| *Myoviridae* | unknown | *Biquartavirus* | *Aeromonas virus 44RR2* | AY375531 | France | 173.591 |
| *Myoviridae* | unknown | *Arv1virus* | *Arthrobacter virus ArV1* | KM879463 | Lithuania | 71.2 |
| *Myoviridae* | unknown | *Marthavirus* | *Arthrobacter virus Martha* | KU160656 | USA | 51.027 |
| *Myoviridae* | unknown | *Agatevirus* | *Bacillus virus Agate* | JX238501 | Poland | 149.844 |
| *Myoviridae* | unknown | *B4virus* | *Bacillus virus B4* | JN790865 | South Korea | 162.956 |
| *Myoviridae* | unknown | *Bastillevirus* | *Bacillus virus Bastille* | JF966203 | Switzerland | 153.962 |
| *Myoviridae* | unknown | *Bc431virus* | *Bacillus virus Bc431* | JX094431.1 | Egypt | 158.621 |
| *Myoviridae* | unknown | *Cp51virus* | *Bacillus virus CP51* | KF554508.2 | Switzerland | 138.658 |
| *Myoviridae* | unknown | *Nit1virus* | *Bacillus virus NIT1* | AP013029 | Japan | 155.631 |
| *Myoviridae* | unknown | *Wphvirus* | *Bacillus virus WPh* | HM144387 | Switzerland | 156.897 |
| *Myoviridae* | unknown | *Abouovirus* | *Brevibacillus virus Abouo* | KC595517 | USA | 45.552 |
| *Myoviridae* | unknown | *Jimmervirus* | *Brevibacillus virus Jimmer* | KC595514 | USA | 54.312 |
| *Myoviridae* | unknown | *Cd119virus* | *Clostridium virus phiCD119* | NC_007917 | USA | 53.325 |
| *Myoviridae* | unknown | *Cd119virus* | *Clostridium virus phiCD27* | NC_009231 | USA | 56.538 |
| *Myoviridae* | unknown | *Msw3virus* | *Edwardsiella virus MSW3* | AB767244 | Japan | 42.746 |
| *Myoviridae* | unknown | *Agrican357virus* | *Erwinia virus Ea35-70* | KF806589 | Canada | 271.084 |
| *Myoviridae* | unknown | *Cvm10virus* | *Escherichia virus CVM10* | GU903191 | USA | 41.666 |
| *Myoviridae* | unknown | *Hapunavirus* | *Halomonas virus HAP1* | NC_010342 | USA | 39.245 |
| *Myoviridae* | unknown | *Kp15virus* | *Klebsiella virus KP15* | GU295964 | Poland | 174.436 |
| *Myoviridae* | unknown | *Kpp10virus* | *Pseudomonas virus KPP10* | AB472900.2 | Japan | 88.322 |
| *Myoviridae* | unknown | *Pakpunavirus* | *Pseudomonas virus PAKP1* | KC862297 | France | 93.198 |
| *Myoviridae* | unknown | *Pbunavirus* | *Pseudomonas virus PB1* | NC_011810 | Canada | 65.764 |
| *Myoviridae* | unknown | *Rheph4virus* | *Rhizobium virus RHEph4* | JX483876 | Mexico | 53.018 |
| *Myoviridae* | unknown | *Spn3virus* | *Salmonella virus SPN3US* | JN641803 | South Korea | 240.413 |
| *Myoviridae* | unknown | *M12virus* | *Sinorhizobium virus M12* | KF381361 | USA | 194.701 |
| *Myoviridae* | unknown | *Sep1virus* | *Staphylococcus virus SEP1* | KF021268 | Portugal | 139.928 |
| *Myoviridae* | unknown | *Schizot4virus* | *Vibrio virus KVP40* | NC_005083 | USA | 244.834 |
| *Myoviridae* | unknown | *Vhmlvirus* | *Vibrio virus VHML* | AY133112 | Australia | 43.198 |
| *Myoviridae* | unknown | *Svunavirus* | *Geobacillus virus GBSV1* | NC_008376 | China | 34.683 |
| *Myoviridae* | unknown | *Machinavirus* | *Erwinia virus Machina* | KX397370 | USA | 241.654 |
| *Myoviridae* | unknown | *Bcep78virus* | *Xanthomonas virus OP2* | NC_007710 | Japan | 46.643 |
| *Myoviridae* | unknown | *Bcep78virus* | *Burkholderia virus BcepNY3* | NC_009604 | USA | 47.382 |
| *Myoviridae* | unknown | *Bxz1virus* | *Mycobacterium virus HyRo* | KT281790 | USA | 153.714 |
| *Myoviridae* | unknown | *Tg1virus* | *Yersinia virus TG1* | KP202158 | Canada | 162.101 |
| *Myoviridae* | *Tevenvirinae* | *Moonvirus* | *Citrobacter virus Moon* | KM236240 | USA | 170.341 |
| *Myoviridae* | *Tevenvirinae* | *Cc31virus* | *Escherichia virus CC31* | GU323318 | USA | 165.54 |
| *Myoviridae* | *Tevenvirinae* | *Cc31virus* | *Enterobacter virus PG7* | NC_023561 | China | 173.276 |
| *Myoviridae* | *Tevenvirinae* | *Js98virus* | *Escherichia virus JS98* | NC_010105 | Switzerland | 170.523 |
| *Myoviridae* | *Tevenvirinae* | *Rb49virus* | *Escherichia virus RB49* | NC_005066 | France | 164.018 |
| *Myoviridae* | *Tevenvirinae* | *Rb69virus* | *Escherichia virus RB69* | NC_004928 | USA | 167.56 |
| *Myoviridae* | *Tevenvirinae* | *T4virus* | *Escherichia virus T4* | NC_000866 | USA | 168.903 |
| *Myoviridae* | *Tevenvirinae* | *Jd18virus* | *Klebsiella virus JD18* | KT239446 | China | 166.313 |
| *Myoviridae* | *Tevenvirinae* | *S16virus* | *Salmonella virus S16* | HQ331142 | Switzerland | 160.221 |
| *Myoviridae* | *Tevenvirinae* | *Sp18virus* | *Shigella virus SP18* | GQ981382 | Republic of Korea | 170.605 |
| *Myoviridae* | *Tevenvirinae* | *Sp18virus* | *Escherichia virus VR26* | KP007362 | Lithuania | 171.541 |
| *Myoviridae* | *Peduovirinae* | *P2virus* | *Escherichia virus P2* | NC_001895 | Sweden | 33.593 |
| *Myoviridae* | *Peduovirinae* | *P2virus* | *Escherichia virus 186* | NC_001317 | Australia | 30.624 |
| *Myoviridae* | *Peduovirinae* | *Hp1virus* | *Haemophilus virus HP1* | NC_001697 | USA | 32.355 |
| *Myoviridae* | *Spounavirinae* | *Spo1virus* | *Bacillus virus SPO1* | NC_011421 | USA | 132.562 |
| *Myoviridae* | *Spounavirinae* | *Tsarbombavirus* | *Bacillus virus TsarBomba* | KT224359 | USA | 162.486 |
| *Myoviridae* | *Spounavirinae* | *P100virus* | *Listeria virus P100* | DQ004855 | Switzerland | 131.384 |
| *Myoviridae* | *Spounavirinae* | *P100virus* | *Listeria virus A511* | NC_009811 | Switzerland | 137.619 |
| *Myoviridae* | *Spounavirinae* | *Kayvirus* | *Staphylococcus virus K* | NC_005880 | USA | 148.317 |
| *Myoviridae* | *Spounavirinae* | *Silviavirus* | *Staphylococcus phage vB_SauM_Romulus* | JX846613 | Belgium | 131.332 |
| *Myoviridae* | *Spounavirinae* | *Silviavirus* | *Staphylococcus virus Stau2* | KP881332 | Taiwan | 133.798 |
| *Myoviridae* | *Vequintavirinae* | *V5virus* | *Escherichia virus V5* | DQ832317 | Canada | 137.947 |
| *Myoviridae* | *Vequintavirinae* | *Cr3virus* | *Cronobacter virus CR3* | JQ691612 | South Korea | 149.273 |
| *Myoviridae* | *Vequintavirinae* | *Se1virus* | *Salmonella virus SE1* | GU070616 | Portugal | 145.964 |
| *Myoviridae* | *Vequintavirinae* | *Se1virus* | *Escherichia virus 4MG* | KF550303 | Korea | 148.567 |
| *Myoviridae* | *Eucampyvirinae* | *Cp220virus* | *Campylobacter virus CP220* | FN667788 | United Kingdom | 177.534 |
| *Myoviridae* | *Eucampyvirinae* | *Cp8virus* | *Campylobacter virus CP30A* | JX569801 | UK | 133.572 |
| *Myoviridae* | *Ounavirinae* | *Mooglevirus* | *Citrobacter virus Moogle* | KM236239 | USA | 87.999 |
| *Myoviridae* | *Ounavirinae* | *Ea214virus* | *Erwinia virus Ea214* | EU710883 | Canada | 84.576 |
| *Myoviridae* | *Ounavirinae* | *Ea214virus* | *Erwinia virus M7* | HQ728263 | Switzerland | 84.694 |
| *Myoviridae* | *Ounavirinae* | *Suspvirus* | *Escherichia virus SUSP1* | KT454805 | USA | 90.743 |
| *Myoviridae* | *Ounavirinae* | *Felixo1virus* | *Salmonella virus Mushroom* | KP143762 | USA | 87.709 |
| *Myoviridae* | *Ounavirinae* | *Felixo1virus* | *Salmonella virus FelixO1* | AF320576 | USA | 86.155 |
