# Supplementary material for: Application of a Novel Phage LPSEYT for Biological Control of Salmonella in Foods
Source: Microorganisms. 2020 Mar 12;8(3):400. doi: 10.3390/microorganisms8030400 (PMC7142823; doi:10.3390/microorganisms8030400)
Supplement: Supplementary file 1 [file microorganisms-08-00400-s001.zip › Supplementary figure 1.docx]

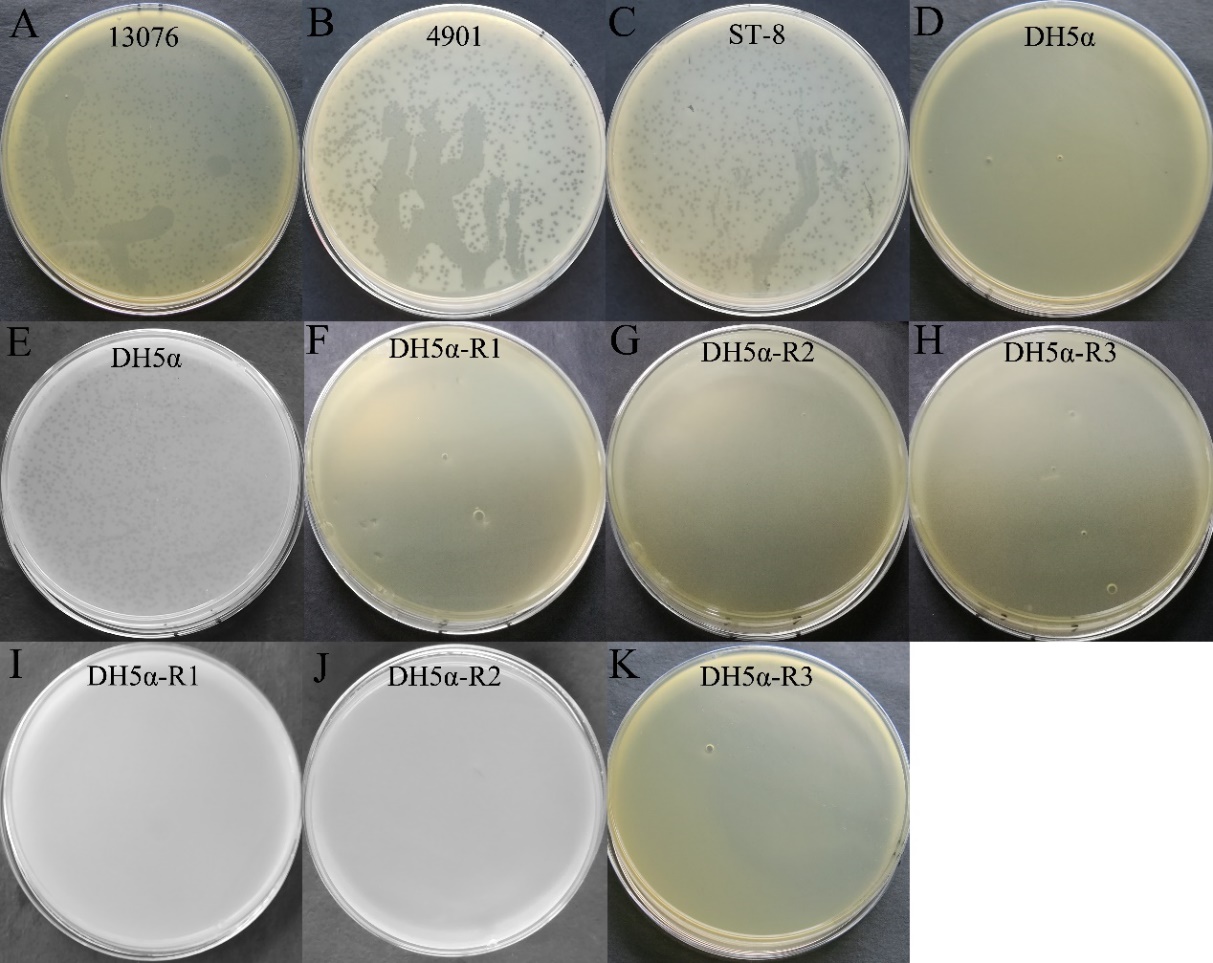


**Supplementary Figure 1.** Verification of the virulent phage LPSEYT. Induced by mitomycin C, (A) *S.* Enteritidis ATCC 13076, (B) *S.* Enteritidis SGSC 4901, (C) *S.* Typhimurium ST-8 were found to contain lysogenic phage, while (D) *E. coli* DH5α was not. (E) The result confirmed that DH5α was the host of phage LPSEYT and can be used for generation of phage-resistant host strain and phage induction experiment. Three representative phage-resistant DH5α strains, namely (F) DH5α-R1, (G) DH5α-R2, and (H) DH5α-R3, were confirmed to be phage resistant. Three phage-resistant strains were induced with mitomycin C respectively, and no phages were detected by the double-layer agar plate method (I: DH5α-R1, J: DH5α-R2, K: DH5α-R3).
